# Porosity Effect of Polystyrene Membranes on Desalination Performance: A Combined Experimental and Numerical Heat and Mass Transfer Study in Direct Contact Membrane Distillation

**DOI:** 10.3390/polym15081821

**Published:** 2023-04-08

**Authors:** Haneen Abdelrazeq, Majeda Khraisheh

**Affiliations:** Department of Chemical Engineering, College of Engineering, Qatar University, Doha P.O. Box 2713, Qatar; ha082881@student.qu.edu.qa

**Keywords:** polystyrene membranes, heat transfer, mass transfer, thermal efficiency, evaporation efficiency

## Abstract

Membrane distillation (MD) is a thermal-based membrane operation with high potential for use in the treatment of aqueous streams. In this study, the linear relationship between the permeate flux and the bulk feed temperature for different electrospun polystyrene membranes is discussed. The dynamics of combined heat and mass transfer mechanisms across different membrane porosities of 77%, 89%, and 94%, each with different thicknesses, are examined. The main results for the effect of porosity with respect to the thermal efficiency and evaporation efficiency of the DCMD system are reported for electrospun polystyrene membranes. A 14.6% increase in thermal efficiency was noted for a 15% increase in membrane porosity. Meanwhile, a 15.6% rise in porosity resulted in a 5% increase in evaporation efficiency. A mathematical validation along with computational predictions is presented and interlinked with the maximum thermal and evaporation efficiencies for the surface membrane temperatures at the feed and temperature boundary regions. This work helps to further understand the interlinked correlations of the surface membrane temperatures at the feed and temperature boundary regions with respect to the change in membrane porosity.

## 1. Introduction

Water desalination is key in the production of fresh water by eliminating undesirable particles from salty water. The seawater stream is mainly the feed that is heated to the point where vapor molecules transfer through the pores of the membrane and condense on the permeate side. Reverse osmosis (RO) is an economically widely used technology that is mostly used for desalinating brine at levels close to those of seawater (<45,000 mg/L) [1,2]. Unfortunately, RO remains an energy-intensive technology [3]. When it comes to energy constraints, MD processes require an energy range of 120.6–1701.8 kWh/m^3^ for heating the feedwater compared to only 2.5–7.0 kWh/m^3^ for the RO process. This huge energy requirement hinders the commercialization potential of advanced MD systems. From an industrial viewpoint, the desalination and treatment of high-salinity brines are inherently energy-intensive [4]. Particularly in MD processes, due to latent heat needed for the evaporation of the feed, the energy requirement significantly increases. The criteria for evaluating the energy performance of an MD system are divided into two main parts: (i) standard measures directly related to the fundamentals of the system, and (ii) developed measures based on the specificity of the employed system [5,6]. Furthermore, combinations of desirable MD features, such as modularity and scalability, have led to a number of simulation and experimental investigations focusing on innovative MD processes for the treatment of hypersaline feeds, especially at larger scales [7].

Membrane distillation (MD) is one of the most widely used thermally driven techniques resulting from a combined heat and mass transfer mechanism through a hydrophobic microporous membrane [8,9]. Generally, saline water is purified in MD, where vapor passes across the pores of the hydrophobic membranes from the hot feed side to the cold permeate side. The temperature slope occurring between the liquid and vapor interfaces at the entrance of the membrane pores results in the driving force of MD [10,11,12].

**Figure 1:**
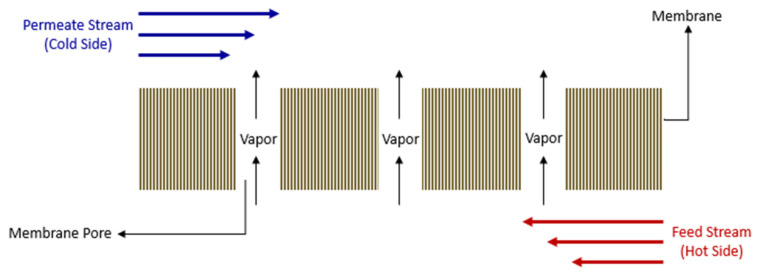
Illustration of direct contact membrane distillation.

In direct contact membrane distillation (DCMD), the two liquid streams come into direct contact with the membrane from both sides, as illustrated in Figure 1. A complex heat transfer system occurs that is limited to the membrane thickness in DCMD. Therefore, the system’s permeate impacts the mass transfer coefficients in the two counter-current streams [13,14,15,16]. The feed temperature in the system and the type of membranes used in DCMD must be optimized in such a way that condensation is prevented within the pores of the membranes. Furthermore, the type of polymer membrane material employed in DCMD systems depends mainly on the specific requirements and constraints of the application, such as the type of feed solution being treated, the desired separation performance, the operating conditions, and the cost.

To date, commercial membranes have been common contributors to the MD process as, until now, there is no commercial membrane that is specially designed for MD [17]. Nonetheless, recent review articles reported valuable summaries on water purification technologies and porous membrane materials, with reference to membrane properties in MD [18,19,20,21,22,23]. For instance, Yang et al. [24] provided an excellent overview of new polymeric membranes and compared different classes of polymeric membranes for water purification applications. The research group determined that the fouling resistance and permeability can be improved by the addition of a microporous support. The most common types of polymers used are polytetrafluoroethylene (PTFE), polyvinylidene fluoride (PVDF), polypropylene (PP), and polystyrene (PS) [25,26,27,28,29,30,31,32,33,34,35,36]. Polystyrene is abundant and can reach up to several million tons of annual production [17]. It has also been distinguished for its competitive cost compared with PVDF and PTFE [37].

Polystyrene (PS) membranes are commonly used in DCMD processes. They are known for their good thermal stability, chemical resistance, and mechanical strength, which makes them appropriate for a wide variety of MD applications [13,38]. An excellent technique by which the membrane’s properties can be controlled is called electrospinning. This fabrication method produces nanofibrous membranes from polystyrene and other polymeric materials with controlled porosity and fiber diameters and high hydrophobicity, making it a very good candidate for applications in membrane distillation systems.

In DCMD, heat inside the feed and permeate solutions is transferred in the forms of sensible and latent heat, and convective heat. Therefore, it is essential to consider the mechanisms of heat conduction and heat convection within the study of heat evolution inside a DCMD module. The pores in the membrane allow the water vapor to pass through while blocking the impurities. The size and distribution of the pores can have a significant effect on the mass transfer properties of the membrane. If the pores in the membrane are too small, the mass transfer rate will be limited because the water vapor will have difficulty passing through the pores. On the other hand, if the pores are too large, the membrane may be less effective at separating impurities from the water vapor. Figure 1 illustrates a schematic diagram of a counter-current DCMD module. In addition to the pore size, the thickness also plays a key role in impacting the heat transfer mechanisms at the membrane boundary where the feed and the permeate solutions are affected by each other [39]. Porosity can affect mass transfer properties by influencing the surface area of the membrane available for mass transfer, and it can also affect the permeability of the membrane. Therefore, the selection of a membrane with the appropriate pore size, thickness, and porosity is key to optimizing the mass and heat transfer properties of the system.

Existing studies in the literature majorly focused on developing optimized models with respect to changes in process conditions and experimental parameters. Eleiwi et al. proposed a dynamic model for the DCMD process, which considers the time evolution of the heat and mass transfer mechanisms throughout the feed and the permeate solutions [39]. Kuang et al. studied the variation in the mass flow rate and concentration in DCMD using computational fluid dynamics, where the water production increased by 28.3% using a 1 mm long module [40]. Elmarghany et al. conducted a thermal investigation for a similar system, where it was shown that increasing the feed temperature negatively affected the thermal performance due to heat loss from the membrane cell [41]. This paper utilizes numerical predictions to optimize the membrane surface temperature based on controlled process parameters and to provide insights into how the porous structure of polystyrene membranes affects the efficiency of DCMD systems at a larger scale.

Several efforts have been made to address the effect of varying process conditions on the heat and mass transfer. Zhang et al. [42] presented a novel model discussing the effect of varying feed salinity (3.5 wt%, 7.5 wt%, and 20 wt%) on hollow-fiber DCMD using MATLAB. Although the proposed simulation could not precisely predict the surface temperatures at higher feed concentrations, the highest water recovery of 86.8% was achieved in seawater compared with that in desalination brine, with 72.1%, at a feed temperature of 50 °C. Similarly, de Sampaio [43] studied the modeling of DCMD consisting of shell and hollow fiber tubes for a desalination plant utilizing heat recovery. For a single DCMD unit, mass and energy conservation and thermodynamic modeling were included. The data showed good agreement with the experimental values in the literature. Additionally, Ansari et al. [44] tested commercial membranes in DCMD, and their results showed a less than 7% deviation with respect to computational modeling data. The research findings showed a 2.3-fold improvement in water flux when the Reynolds number was increased from 80 to 1600, along with a 23% increase in thermal efficiency when the membrane porosity was increased from 40% to 70%. Moreover, other research groups focused on addressing the fouling phenomenon in DCMD using a cake filtration theory to signify the reduction in flux due to inorganic fouling [45]. This led to a significant enhancement in the overall efficiency of MD and a reduction in the number of membrane washing cycles. Nonetheless, it is also important to understand the impact of porosity on heat and mass transfer in efforts to optimize the design of direct contact membrane distillation systems and improve their performance. Due to the scarcity of studies that attempt to study the relationship between the porosity effect and mass flux and its effect on the mass and heat transfer with respect to the variable operating conditions of pilot-scale MD systems, this study helps to further understand the mechanism of heat flux flow through the membrane pores and offers a numerical validation along with computational predictions that aim to find the optimum surface temperatures that are interlinked with the maximum thermal and evaporation efficiencies at the thermal boundary regions at a pilot scale. Moreover, to the best of our knowledge, the current literature has not yet explored the desalination potential of electrospun polystyrene membranes in DCMD at a pilot scale The scope of this work is limited to the optimization of membrane surface temperatures based on controlled process parameters, providing insights into how the porous structure of polystyrene membranes affects the efficiency of a pilot DCMD unit. Moreover, the theoretical and predicative results are validated with laboratory experiments, without considering inorganic fouling.

## 2. Experimental

### 2.1. Materials

Polystyrene pellets (PS, Mw = 192,000, CAS Number: 9003-53-6) and N,N-dimethyl formamide (DMF, CAS Number: 68-12-2, 99.8% purity) were purchased from Sigma Aldrich, Saint Louis, MO, USA and used as is.

### 2.2. Membrane Synthesis

A custom-made electrospinning system was used for membrane fabrication in this work (Figure 1). An amount of 20 g of polystyrene (molecular weight: 192,000) was dissolved in 60 mL DMF and 40.0 mL acetone. PS and DMF were first set to stir in a beaker. This was followed by continuous stirring for acetone at room temperature for up to 24 h to ensure the polymers dissolved. Then, 0.1%, 0.5%, and 1% of PTFE powder were added to the synthesized polystyrene membranes M1, M2, M3, and M4. The spinning parameters used were as follows: volume of polymer solution, 10 mL; voltage, 14 kV; distance between the needle and collector, 15 cm; needle diameter, 20-gauge needle; flow rate, 6 mL/h; and drum RPM, 340 RPM. The fabricated membranes were then left in a vacuum oven overnight to eliminate any residuals. Afterwards, they were cold-pressed at 1 Ton for 1 min. The electrospun membranes are listed in Table 1, where sample masses of 0.1629 g, 0.1420 g, 0.1342 g, and 0.0510 g correspond to M1, M2, M3, and M4, respectively.

### 2.3. DCMD Pilot-Scale Investigation

A pilot unit was used for all the experimental tests using the electrospun flat-sheet polystyrene membranes. An experimental feed/permeate flow rate of 40 L/h was used in the predictions to evaluate the optimum permeate flux at fixed inlet feed and permeate temperatures of 70 °C and 20 °C, respectively. The feed spacer was the same for all experiments, and its influence on concentration polarization was not investigated. A similar pilot unit was included in our previous study for testing commercial polyethylene membranes [46].

## 3. Mathematical Modeling

### 3.1. Heat Transfer

#### 3.1.1. Heat Transfer from the Feed Side to the Surface of the Electrospun Membrane

Convection is used to transfer heat through the feed boundary layer, and Newton’s law of cooling governs this process by the following equation:

(1) $$Q_{f}=h_{f}\left(T_{b,f}-T_{m,f}\right)$$

where $Q_{f}\;$ is the convective heat flux, $h_{f}$ is the boundary layer heat transfer coefficient on the membrane’s feed side, and $T_{b,f}$ and $T_{m,f}\;$ are the average feed temperatures for the bulk and surface of the membrane from the feed side, respectively. The transfer of heat across the membrane can be categorized into two segments: the first is the transfer of heat through the membrane by conduction, which includes the polymer matrix and pores filled with gas; the second is the transfer of heat through the membrane by the latent heat of water vapor movement.

#### 3.1.2. Second Stage: Heat Transfer through the Membrane Layer

The conducted heat transfer across the membrane (*Q_C_*) is added to the evaporative mass flow (*Q_v_*) through the membrane pores to obtain the total heat flux across the membrane (*Q_m_*).

(2) $$Q_{c}=\frac{k_{m}}{\delta }\left(T_{m,f}-T_{m,p}\right)$$

(3) $$Q_{v}=J_{w}\Delta H_{v}$$

The enthalpy of the water $(\Delta H_{v})\;$ can be calculated using the following equation:

(4) $$\Delta H_{v}=\left(\left(1.7535*T_{m,f}\right)+2024.3\right)$$

The effective thermal conductivity of the membrane (*k_m_*) is equal to the product of the thermal conductivity of the solid membrane (*k_mem_*) and the thermal conductivity of the membrane gas (*k_gas_*) (air and water vapor).

(5) $$k_{m}={\left(\left(\frac{\epsilon }{k_{gas}}\right)+\left(\frac{1-\epsilon }{k_{mem}}\right)\right)}^{-1}$$

The total heat flux across the membrane (*Q_m_*) can be described as the following:

(6) $$Q_{m}=Q_{c}+Q_{v}=\;h_{m}\left(T_{m,f}-T_{m,p}\right)+J_{w}\Delta H_{v}$$

#### 3.1.3. Third Stage: Heat Transfer from the Membrane Surface to the Permeate Stream

Convection is used to transmit heat across the boundary layer from the permeate-side membrane surface to the permeate bulk. The permeate heat flux, $Q_{p}$, depends on the permeate heat transfer coefficient ($h_{p}$) and temperature difference between the bulk permeate temperature ($T_{b,p})$ and the interfacial membrane temperature ($T_{m,p}$) on the permeate side. In this work, the DCMD process is assumed to be a steady-state process in order to calculate the surface temperature on both the feed and permeate sides of the membrane. The overall heat transfer fluxes of the feed, membrane, and permeate sides of the module are assumed to be under steady-state conditions ($Q_{f}=Q_{m}=Q_{p}$).

(7) $$Q_{p}=h_{p}\left(T_{m,p}-T_{b,p}\right)$$

In the DCMD process, the vapor pressure difference arising from the temperature difference between the two surfaces of the membrane is the driving force for water vapor transfer across the membrane. The temperature difference between $T_{m,f}$ (the membrane/feed interface) and $T_{m,p}$ (the membrane/permeate interface) is the driving force for water vapor transfer through the pores of the membrane. However, one of the limitations in DCMD systems is the change in the membrane/interface temperature with respect to the bulk temperature in the process. This occurs due to heat lost from the feed stream side of the membrane surface and heat gained from the permeate stream side of the membrane surface. $T_{m,f}$ and $T_{m,p}$ are calculated using the following equations:

(8) $$T_{m,f}=\frac{k_{m}\left(T_{b,p}+\frac{h_{f}}{h_{p}}T_{b,f}\right)+\delta \left(h_{f}T_{b,f}-J_{w}\Delta H_{v}\right)}{k_{m}+h_{f}\left(\delta +\frac{k_{m}}{h_{p}}\right)}$$

(9) $$T_{m,p}=\frac{k_{m}\left(T_{b,f}+\frac{h_{p}}{h_{f}}T_{b,p}\right)+\delta \left(h_{p}T_{b,f}+J_{w}\Delta H_{v}\right)}{k_{m}+h_{p}\left(\delta +\frac{k_{m}}{h_{f}}\right)}$$

In this work, the DCMD process is assumed to be a steady-state process in order to calculate the surface temperature of both the feed and permeate sides of the membrane. A number of assumptions were made to assess the significance of the different heat transfer mechanisms using the pilot DCMD system: for example, the operating conditions are in a steady state; there is negligible heat loss; the membrane pores have uniform sizes; the water has constant physical properties; the water flow is laminar in the x-direction; and there is a constant total pressure of 1 atm. As such, the heat balance guarantees that the three consecutive heat transfer methods satisfy the following equation:

(10) $$Q_{f}=Q_{m}=Q_{p}$$

After a certain period of time, the concentration polarization in the desalination process influences the transfer as a result of salt molecules building up on the membrane surface. The ratio of the solute concentration on the feed membrane surface (*C_m,f_*) to the concentration of the feed bulk (*C_b,f_*) is known as the concentration polarization coefficient (ϕ):

(11) $$\phi =\frac{C_{m,f}}{C_{b,f\;}}$$

(12) $$C_{m,f}=C_{b,f}*\exp\left(\frac{J_{w}}{k_{s}*\rho_{b,f}}\right)$$

where $\rho_{b,f}$ is the density of the feed flow, and $k_{s}$ represents the solute mass tranfer coefficient as follows:

(13) $$k_{s}=Sh*\frac{D_{e}}{D_{h}}$$

where *D_h_* is the hydraulic diameter of the hot channel, and *Sh* is the Sherwood number, which is determined using the Graetz–Leveque equation for laminar flow:

(14) $$Sh=1.86{\left(Re*Sc*\frac{D_{h}}{L}\right)}^{\frac{1}{3}}$$

In the following equations, *Sc* represents the Schmidt numbers, *Re* represents the Reynolds number, and *Pr* represents the Prandtl number:

(15) $$Sc=\frac{\mu_{mf}}{\rho_{b,f}*D_{e}}$$

(16) $$h=\frac{Nu*k}{D_{h}}$$

(17) $$\mathrm{Pr}=\frac{v}{\alpha }=\frac{\mu *c_{p}}{k}\;$$

where *k* is the average thermal conductivity of the fluid on the membrane feed side, and *Nu* is the Nusselt number, which is determined using the equation shown below. For a flat-plate module and laminar flow (*Re* < 2100), the Nusselt number can be used for both the feed and permeate sides of the membrane using the following equation:

(18) $$Nu=1.86\;{\left(\frac{RePrD_{h}}{L}\right)}^{\frac{1}{3}}$$

### 3.2. Mass Transfer

In the following equation, $J_{w}$ is the permeate mass flux, and *D_e_* is the equivalent diffusion coefficient:

(19) $$J_{w}=D_{e}*\Delta p_{m}=D_{e}*\left(P_{wf}^{0}-P_{wp}^{0}\right)$$

where $P_{wf}^{0}$ and $P_{wp}^{0}$ are the partial pressures of water on the feed and permeate sides of the membrane, respectively:

(20) $$P_{wf}^{0}=\exp\left(23.1964-\frac{3816.44}{T_{mf}-46.13}\right)$$

(21) $$P_{wf}^{0}=\exp\left(23.1964-\frac{3816.44}{T_{mf}-46.13}\right)$$

Considering the effect of salinity in the feed solution, the permeate flux can be represented by the following equation:

(22) $$J_{w}=D_{e}\left(P_{wf}^{0}*\gamma_{wf}*x_{wf}-P_{wp}^{0}\right)$$

For an aqueous solution of NaCl, $\gamma_{wf}$ can be expressed as the following:

(23) $$\gamma_{wf}=1-\left(0.5*x_{NaCl}\right)-\left(10*x_{Nacl}^{2}\right)$$

There are three different types of mechanisms that account for the movement of gases and vapor through porous media, which are the Poiseuille flow model, the molecular diffusion model, and the Knudsen model. The Knudsen flow and molecular diffusion models can be used in DCMD. The trans-membrane hydrostatic pressure is not applied since the feed and permeate solutions are retained inside the membrane module at a constant pressure (about 1.0 atm). The Poiseuille flow in this situation is insignificant. The ratio of Knudsen diffusion to molecular diffusion is used to calculate the combined influence of the molecular and Knudsen diffusions. The governing mechanism in the mass transmission is determined by this ratio. Considering the effect of salinity, the effective, Knudsen, and molecular diffusion coefficients are *D_e_*, *D_k_*, and *D_m_*, respectively. The following are the mathematical expressions for *D_e_*, *D_k_*, and *D_m_*:

(24) $$D_{e}={\left(\frac{\alpha }{D_{k}}+\frac{1-\alpha }{D_{m}}\right)}^{-1}$$

(25) $$D_{k}={\left(\frac{3*\delta *\tau }{2*\epsilon *d_{pore}}*{\left(\frac{\pi *R*T_{m}}{8*Mol_{w}}\right)}^{0.5}\right)}^{-1}$$

(26) $$D_{m}={\left(\frac{R*T_{m}*\delta *\tau *P_{air,pore}}{Mol_{w}*\epsilon *PD_{w,a}}\right)}^{-1}$$

The following expression, where $PD_{w,a}$ can be employed in the temperature range of 273–373 K, is used to compute the value for water–air, and *T_m_* is the mean temperature across the membrane surfaces:

(27) $$PD_{w,a}=1.895*10^{-5}*T_{m}^{2.072}$$

(28) $$P_{pore}=\frac{P_{f}+P_{p}}{2}$$

(29) $$T_{m}=\frac{T_{mf}+T_{mp}}{2}$$

The fictitious route across the membrane is frequently related to the membrane porosity ϵ, as in the Mackie–Meares equation, and τ is the membrane thickness, which is frequently constant.

(30) $$\tau =\frac{1}{\epsilon }$$

**Figure 2:**
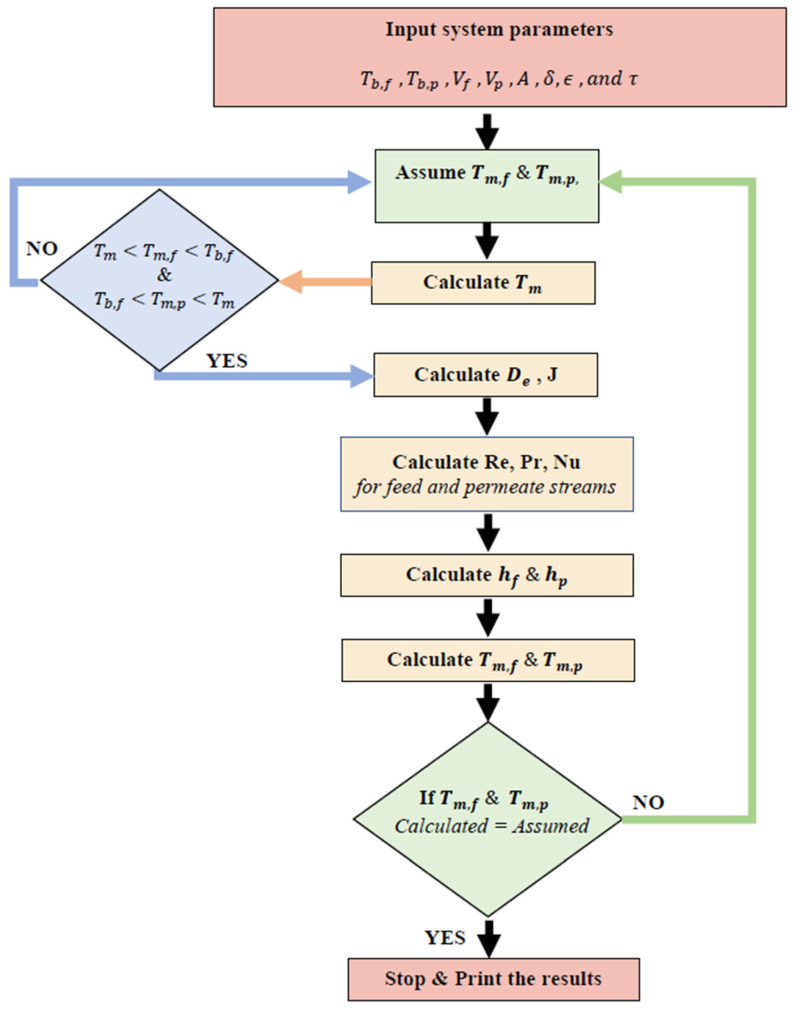
Flowchart for numerical optimization model in direct contact membrane distillation.

The steps followed for calculating the theoretical model in the current DCMD system are depicted in Figure 2.

## 4. Results and Discussion

The accuracy of the model can be enhanced through artificial intelligence and machine learning by utilizing optimization software. Therefore, the data were simulated using Python to find the optimum conditions using the concept of iteration. Most simulation models are tested on the basis of experimental work [12,13]. Hence, DCMD runs were conducted to specifically evaluate the permeate flux under fixed experimental conditions. Hence, to further investigate the effect of varying system conditions on the membrane performance, both theoretical and predictive models were tested on the basis of experimental data. The accuracy of the theoretical mode was enhanced through the utilization of optimization methods in Python. The data were simulated using Python to find the optimum conditions using the concept of iteration.

### 4.1. Permeate Flux

As shown in Table 2, the increase in the Teflon percentage from 0.1% to 0.5% led to a slight reduction in the experimental permeate flux by 1.8%. This decrease in the permeate flux is attributed to the increase in membrane thickness from 190 µm to 199 µm. Furthermore, at a fixed porosity of 94%, the flux was enhanced by 2.7% and 0.44% with the addition of 1% for polystyrene membranes with 157 µm and 131 µm thicknesses, respectively. It can be observed that the optimum flux was achieved by the membrane with 14.05 LMH. This value is relatively higher than that of other membranes in DCMD, as listed in Table 3. This is attributed to the presence of PTFE beads that acted as a template for the formation of pores in the membrane, leading to an increase in its porosity. The mechanism of MD involves the application of a temperature gradient across a porous membrane, which creates a difference in vapor pressure on either side of the membrane. Water molecules in the liquid phase evaporate on the warm side of the membrane and diffuse through the pores to the cold side, where they condense into a liquid phase. The process is driven by the difference in vapor pressure across the membrane. The effectiveness of MD systems largely depends on several factors such as the pore size and thickness of the membrane, as well as the temperature and concentration of the saline feed across the membrane. A smaller pore size can reduce the rate of water vapor transport due to the longer diffusion path, which may result in a higher energy consumption to achieve the desired level of water recovery. On the other hand, larger pore sizes can increase the rate of transport and potentially lead to better energy efficiency, but may also increase the risk of wettability.

**Figure 3:**
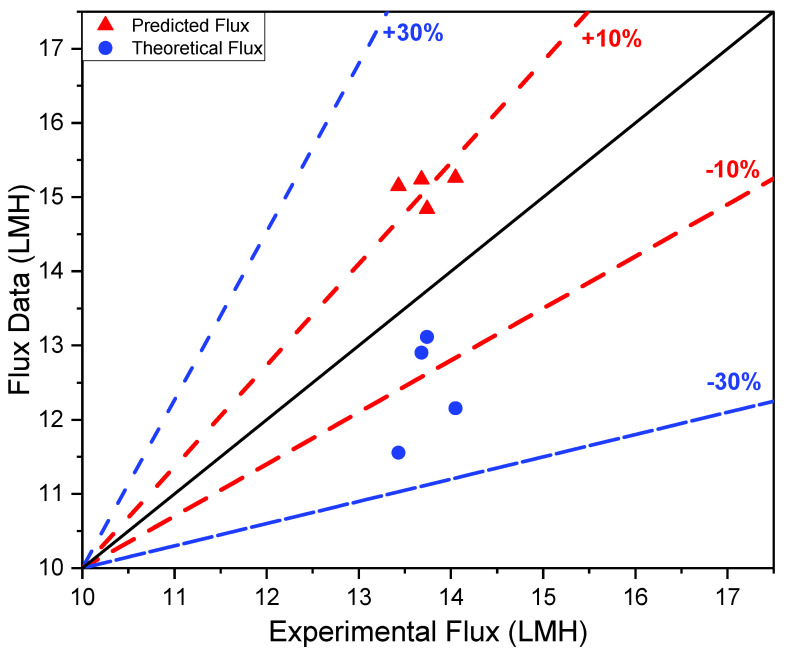
Numerical validation of predicted and theoretical flux with respect to experimental permeate flux for polystyrene membranes in pilot scale DCMD.

Alternatively, a thinner membrane can enhance the heat transfer rate across the membrane, leading to enhanced energy efficiency. However, it may also increase the risk of membrane fouling, which can decrease the overall efficiency of the process. In contrast, a thicker membrane can provide better mechanical strength and durability but may result in lower energy efficiency due to the reduced heat transfer. Therefore, selecting the optimal pore size and membrane thickness requires balancing the trade-offs between membrane durability, energy consumption, and the potential occurrence of membrane wettability in order to achieve an improved desalination performance. Similar to previous studies, an optimized numerical model was used to evaluate the experimental values of the membrane/liquid interface temperatures, the thermal efficiency of the system, and the evaporation efficiency [39,47]. The predicted flux resulted in a similar trend to the experimental values. Nonetheless, the theoretical flux showed a comparatively high error of 13% in relation to that of the experimental flux. This is because the applied theoretical model investigated a wider range of applicability, leading to a higher probability of error in the obtained flux, as presented in Figure 3. For this reason, optimization was performed using Python in order to account for the variation in the surface membrane temperatures on both the feed and permeate sides to accurately predict the flux.

**Figure 4:**
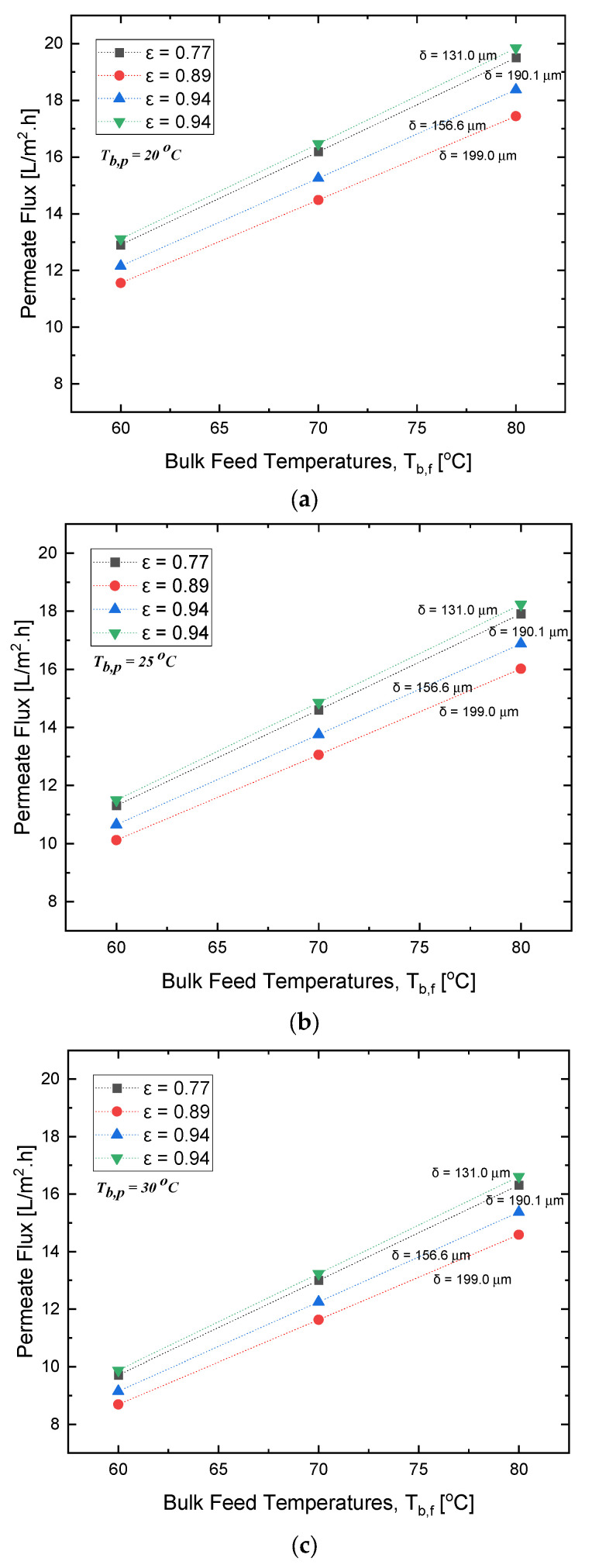
Effect of varying membrane porosity with respect to change in the bulk feed temperature and membrane thickness at a *T*_*b*,*p*_ of (**a**) 20 °C, (**b**) 25 °C, and (**c**) 30 °C.

In the results depicted in Figure 4, it can be observed that there is a linear relationship between the permeate flux and the bulk feed temperature, and that, by looking at the 89% porosity, as the bulk feed temperature increased from 60 °C to 70 °C and from 70 °C to 80 °C, the permeate flux increased from almost 11.9 to 14 LMH, and from 14 to 16.2 LMH, respectively.

Additionally, the results show that there is no direct relationship between the increase in flux and the increase in membrane porosity, as, for example, the lowest porosity does not correspond to the lowest permeate flux, which means that the increase in flux is not proportional to the increase in porosity. The same conclusion can be applied to the relation between the permeate flux and membrane thickness. It is worth mentioning that the lowest experimental flux was observed at the largest thickness of 199 μm, with a porosity of 89%. However, the optimum flux was achieved at the highest porosity, with a slightly lower thickness of 157 μm.

**Table 3:** Comparison of the flux predicted in this work with various experimental performances of different membranes in the literature.

Membrane	Feed Temperature (°C)	Feed Concentration (g/L)	Feed Flow Rate (L/min)	Experimental Flux (LMH)	Ref.
**PVDF**	50	35	0.6	21	[48]
	80	0.45	6	51.5	[49]
**PTFE**	40–90	4.65	0.14–100	55–72	[50]
	60	Seawater	4.5	45.5	[51]
	38	Various	11–22	2–5	[52]
	60	Synthetic brine	0.03	4.85–15.95	[13]
**PTFE-PP**	60	30	0.04	12.2	[53]
**PVDF-PTFE**	60	20	0.5	19	[54]
**PP**	40–60	-	0.5–1.7	5–25	[55]
**PE**	80	3.5	1.5	123	[56]
	70	Synthetic brine	1.2	122.2	[46]
**PS**	60	Synthetic brine	0.03	2.9–11.68	[13]
**PS-PS**	65	7	0.05	8.1	[57]
**PS-AC**	65	7	0.05	6.3	[57]
**PS-PTFE**	60–80	Synthetic brine	1.5	13.68–14.05	Present work
**PS-PTFE**	60–80	15	1.5	Predicted flux 14.84–15.26	Present work

### 4.2. Effect of Porosity on Thermal Efficiency

**Figure 5:**
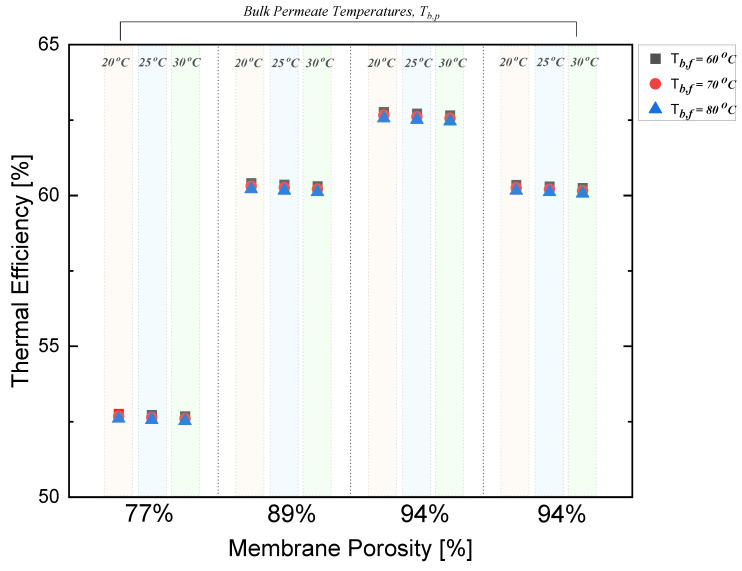
Effect of bulk temperatures on thermal efficiency in DCMD.

At a low membrane porosity of 77%, the membrane showed a minimum thermal efficiency at all bulk feed temperatures. With a 15% increase in membrane porosity, a higher amount of water vapor was transported across the membrane, leading to more heat being exchanged between the two sides of the polystyrene membrane. This resulted in a 14.6% increase in thermal efficiency. Similarly, as depicted in Figure 5, the maximum thermal efficiency of 63% was reached when the membrane porosity was increased by 22%.

Interestingly, at an equal membrane porosity of 94%, increasing the membrane thickness by 14.5% resulted in a thermal efficiency drop by 4%. MD membranes with higher thicknesses have higher thermal resistance, which hinders the heat transfer between both the hot and cold streams. This results in a lower temperature driving force across the membrane, which decreases the rate of mass transfer and reduces the thermal efficiency of the MD process [58]. Moreover, the thermal efficiency of polystyrene membranes in DCMD is majorly affected by membrane fouling. As previously demonstrated, the accumulation of salt particles takes place on the membrane surface, coming from the synthetic feed [13].

### 4.3. Effect of Porosity on Evaporation Efficiency

The membrane porosity was shown to have a significant effect on the evaporation efficiency with respect to changes in the bulk permeate and feed temperatures. A rise in porosity from 77% to 89% resulted in a 5% increase in evaporation efficiency. Higher porosities allow for more efficient evaporation since a larger surface area becomes available for water vapor to pass through. However, as the porosity continued to increase up to 94%, the ability of the membrane to properly reject the dissolved solutes, coming from the feed solution, decreased. This negatively impacted the evaporation efficiency, decreasing it substantially by 5%.

**Figure 6:**
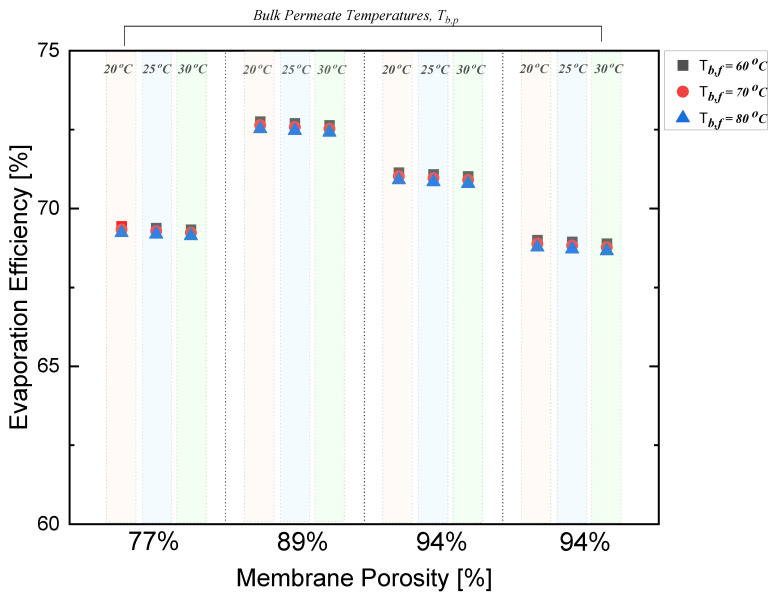
Effect of bulk temperatures on evaporation efficiency in DCMD.

As per the results depicted in Figure 6, as the porosity of the polystyrene membrane increased beyond 89%, the increased surface area for evaporation became offset by the decrease in the solute rejection, resulting in a decrease in evaporation efficiency. In Figure 4a–c, the relationship between membrane porosity, thickness, and bulk permeate temperature is illustrated. Taking the 60 °C bulk feed temperature as an example, the maximum permeate flux can be observed at a porosity of 94% with a thickness of 133 μm; the second highest permeate can be observed at a porosity of 77% with a thickness of 190 μm; the third highest permeate flux can be observed at a porosity of 94% with a thickness of 156.6 μm; and the lowest permeate flux can be observed at a porosity of 89% with a thickness of 199 μm.

With an increase in the bulk feed temperatures, the permeate flux increased by 51% due to the increasing vaporization of the synthetic brine at higher temperatures [38]. This is in accordance with Antoine’s equation, where the vapor pressure exponentially rises with an increase in feed temperature, resulting in a rise in the permeate flux and enhancement of the overall MD process efficiency [59].

**Figure 7:**
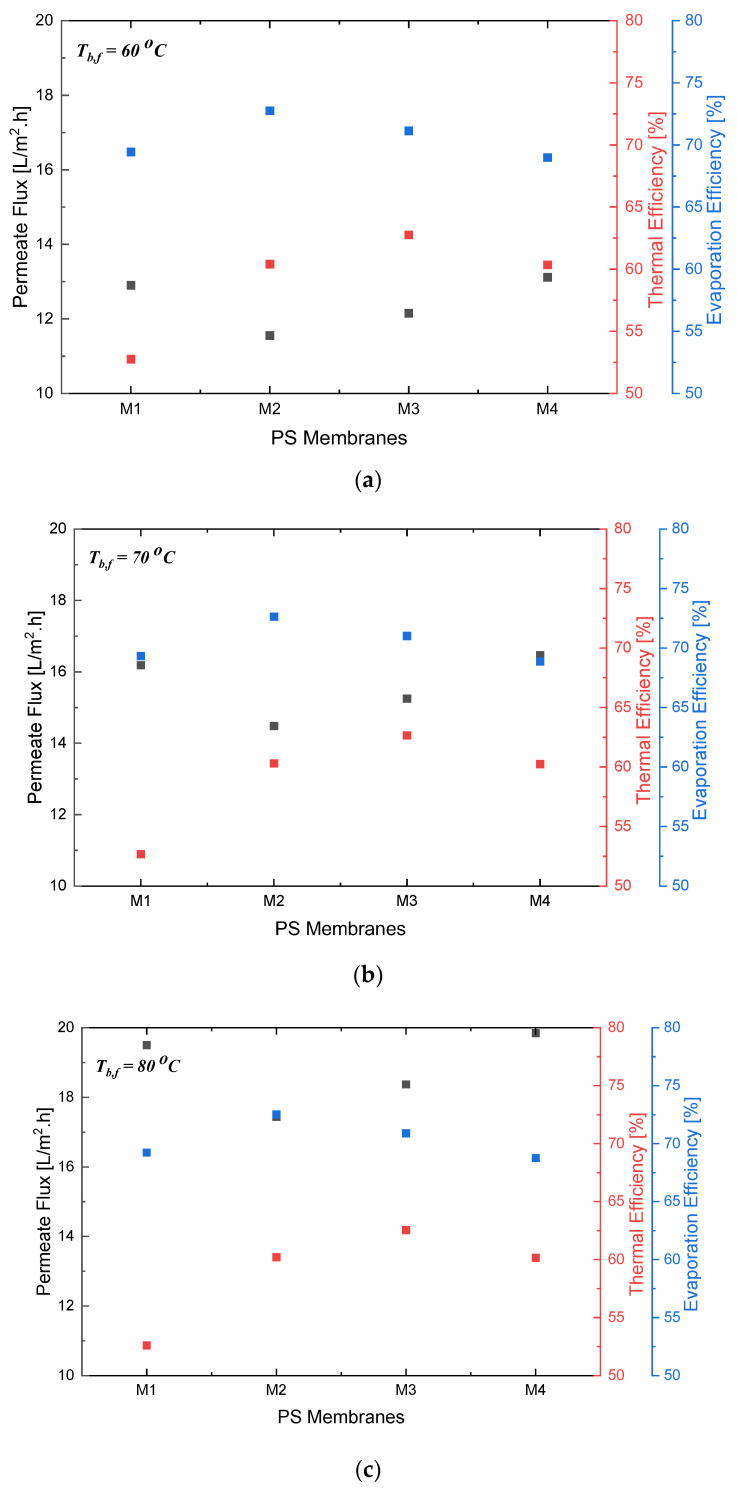
Permeate flux vs. both thermal efficiency and evaporation efficiency of polystyrene membranes at fixed *T*_*b*,*p*_ = 20 °C and changing *T*_*b*,*f*_ from 60 °C to 80 °C in (**a**–**c**), respectively.

Ni et al. investigated the effect of membrane characteristics of different membrane materials [60]. Their study showed that with a decrease in membrane thickness, the permeate flux could be enhanced until a certain limit is reached. This limit is the threshold where the permeate flux is no longer improved. Theoretically, based on the literature, a reduction in thickness results in a continuous increase in the permeate flux. However, this is not the case in experimental investigations. Once the threshold is reached, the efficiency in membrane separation starts to decrease. This trend is depicted in Figure 7. However, Park and Lee investigated the energy efficiency in a pilot-scale DCMD system for hollow-fiber modules [61]. Their study showed that the thermal efficiencies of different MD modules cannot be directly compared in terms of the flux.

**Figure 8:**
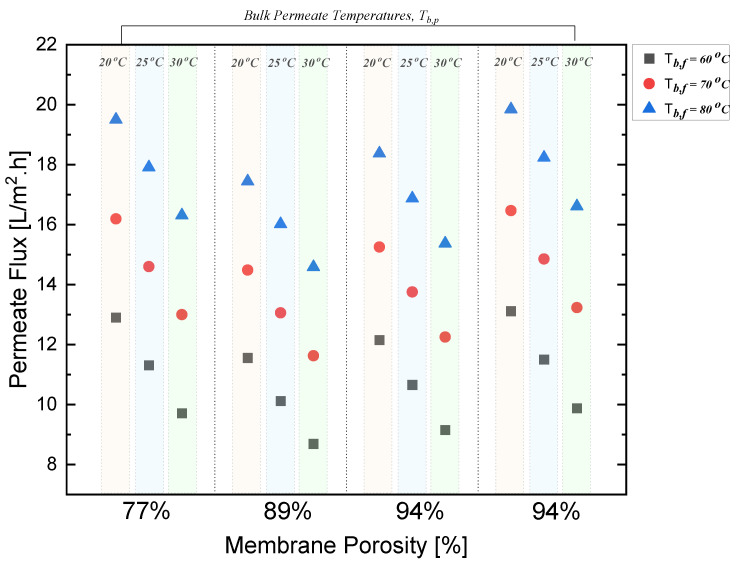
Effect of change in membrane porosity on permeate flux in DCMD.

Figure 8 demonstrates the relationship between the permeate flux and membrane porosity by taking into consideration the change in the bulk permeate and bulk feed temperatures. At a constant porosity of 77% and a constant bulk permeate temperature of 20 °C, it can be observed that the permeate flux increased as the bulk feed temperature increased from 60 °C to 80 °C. The same trend can be observed for the bulk permeate temperatures of 25 °C and 30 °C, and for the rest of porosities as well. At a constant porosity and bulk feed temperature, a decreasing trend with respect to the permeate flux can be observed. Looking at the 77%-porosity membrane, the permeate flux decreased from 30 to 9 LMH when the bulk permeate temperature increased from 20 °C to 30 °C.

At a constant bulk feed and bulk permeate temperature, and with the increase in membrane porosity from 77% to 94%, a non-linear relationship can be observed. The permeate flux first decreased from 12.9 to 11.8 when the porosity increased from 77% to 89%; then, the permeate flux increased from 11.9 to 12.1, and again to 13.1, when the porosity increased from 89% to 94%; afterwards, it stayed constant at 94%.

### 4.4. Effect of Porosity on Mass Transfer Coefficient

**Figure 9:**
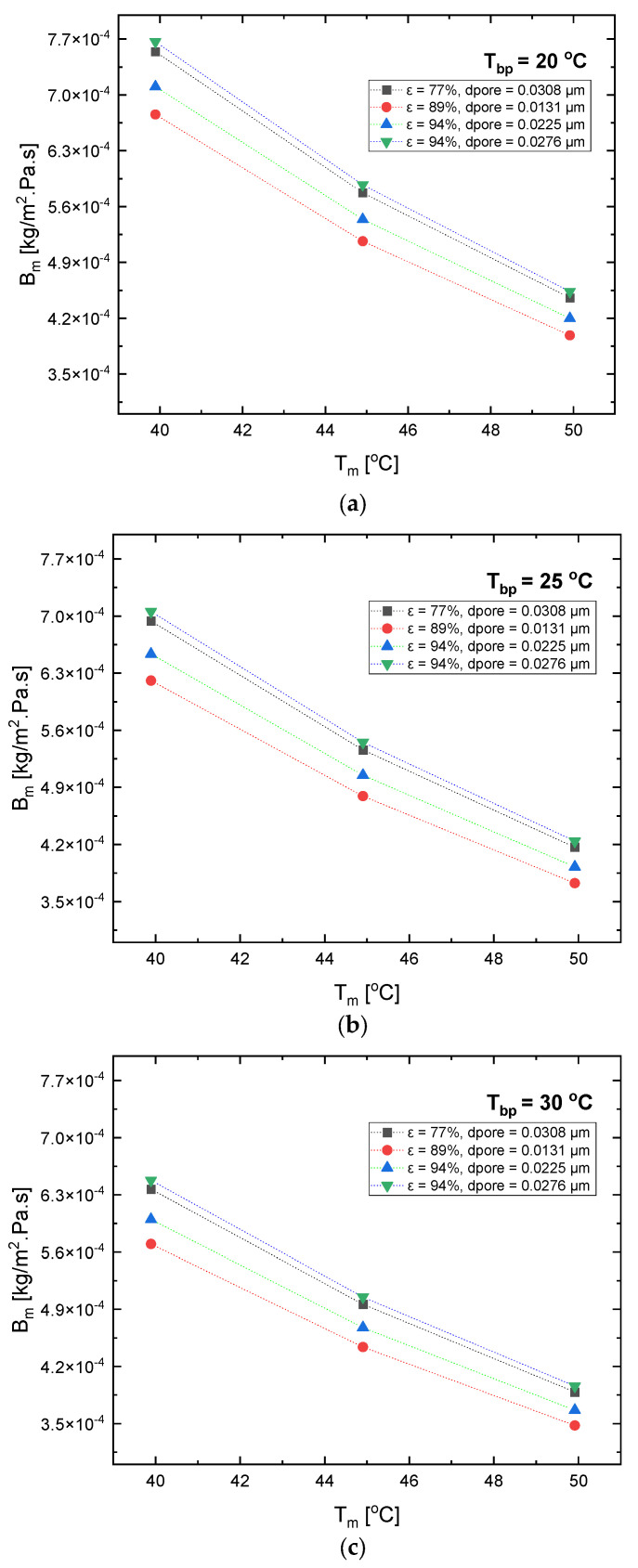
Mass transfer coefficient with respect to change in bulk feed and permeate temperature.

On the other hand, the design and manufacturing of various separation tools are usually quantified by the mass transfer coefficient. The mass transfer coefficient in any MD configuration is significantly dependable upon the membrane’s temperature and characteristics [62]. In Figure 9a, it can be seen that at a constant permeate flux and pore diameter, the mass transfer coefficient values decreased with the increase in the mean average temperature. The maximum mass transfer coefficient value occurred at the highest permeate flux of 94% with a pore diameter of 0.0276 μm, and the lowest mass transfer coefficient occurred at a permeate flux of 89% with a pore diameter of 0.0131 μm. Thus, it can be observed that there is no direct relationship between the increase in the permeate flux, pore size diameter, and variation in the mass transfer coefficient. The same conclusion can be drawn from Figure 9b,c.

As shown in Figure 9b,c, the bulk permeate temperature was increased from 20 °C to 25 °C and 30 °C. Looking at the membrane with a 94% permeate flux and a pore diameter of 0.0276 m at a 40 °C mean average temperature, as the temperature increased from 20 °C to 25 °C and from 25 °C to 30 °C, the mass transfer coefficient decreased from $7.7\times 10^{-4}\;\mathrm{pa}\cdot s$ to $7.0\times 10^{-4}\;\mathrm{pa}\cdot s$, and from $7.0\times 10^{-4}\;\mathrm{pa}\cdot s$ to $6.4\times 10^{-4}\;\mathrm{pa}\cdot s$. This is due to the fact that an increase in temperature can cause the fluid in the pores to become more viscous, which can lower the mass transfer coefficient. This occurs since the fluid becomes more resistant to flow, and also because diffusion through the pores becomes more difficult.

## 5. Conclusions

This study demonstrates the effect of the porous structure of polystyrene membranes on the overall efficiency of a pilot-scale DCMD system. Such insights are difficult to obtain through conventional bench-scale DCMD setups. Supported by the experimental findings, an optimized iterative method was used to minimize the error between the initial estimates of the surface membrane temperature and the actual values, allowing them to predict the temperature accurately in each experiment. The prediction model used in this study was effective in predicting the permeate flux, as the results showed good agreement between the experimental results and the optimization model, with an error between 8 and 13%. The theoretical modeling data showed that the higher the temperature difference between the feed and permeate sides, the greater the vapor pressure difference, resulting in an increased permeate flux, until a threshold is reached, at which point the flux stops improving. The lowest experimental flux was observed at the largest thickness of 199 μm with a porosity of 89%. Additionally, for the 94% membrane porosity, increasing the membrane thickness by 14.5% resulted in a thermal efficiency drop by 4%. The results show that at porosities beyond 89%, the ability of the membrane to efficiently reject the dissolved solutes was reduced. This negatively impacted the evaporation efficiency, decreasing it substantially by 5%. The results obtained in this work can contribute to creating a good basis for future studies on the scalability of PS membranes for potential membrane-based desalination technologies in the industry. Our findings are in line with recent innovative wastewater technologies and are largely accountable for the optimization of industrial MD processes during the treatment of industrial wastewater. Yet, further investigations are still required to understand how heat loss minimization should be carried out experimentally for consideration in larger-scale applications.

## Figures and Tables

**Table 1:** Details of the used PS flat-sheet membranes.

Characteristics	M1	M2	M3	M4
BET surface area (m^2^/g)	30.17	31.21	51.77	57.87
Average pore diameter (4V/A) (μm)	0.0308	0.0131	0.0225	0.0276
Porosity (%)	77	89	94	94

**Table 2:** Experimental, theoretical, and predicted permeate fluxes of polystyrene membranes under controlled parameters of *T*_*b*,*f*_ = 60 °C and *T*_*b*,*p*_ = 20 °C.

Porosity	Thickness (µm)	J_exp_ (LMH)	J_theoretical_ (LMH)	Error (%)	J_predicted_ (LMH)	Error (%)
0.77	190	13.68	12.9032	6	15.24	11
0.89	199	13.43	11.5563	14	15.15	13
0.94	157	14.05	12.1535	13	15.26	9
0.94	131	13.74	13.1158	5	14.84	8

## Data Availability

The data presented in this study are available on request from the corresponding author.

## Nomenclature

*A*	Cross-sectional area [m^2^]
*d_p_*	Pore size [µm]
*d_h_*	Hydraulic diameter [m]
*D*	Diffusion coefficient [m^2^/s]
*h*	Heat transfer coefficient [W/m^2^K]
*H_v_*	Heat of vaporization [kJ/kg]
*J_w_*	Permeate flux [LMH]
*K*	Thermal conductivity [W/mK]
*B_m_*	Mass transfer coefficient [kg/m^2^sPa]
*K_m_*	Membrane thermal conductivity [W/mK]
*K_gas_*	Thermal conductivity of gas filling the pores [W/mK]
*K_mem_*	Thermal conductivity of membrane material [W/mK]
*Kn*	Knudsen number [dimensionless number]
*M_w_*	Molecular weight [g/mol]
*Nu*	Nusselt number [dimensionless number]
*P*	Total pressure [Pa]
*P_m_*	Mean pressure [Pa]
*Pr*	Prandtl number [dimensionless number]
*Q_s_*	Sensible heat transfer [W/m^2^]
*Q_v_*	Latent heat transfer [W/m^2^]
*Qc*	Conduction heat transfer [W/m^2^]
*R*	Gas constant [J/Kmol]
*Re*	Reynolds number [dimensionless number]
*Sc*	Schmidt number [dimensionless number]
*Sh*	Sherwood number [dimensionless number]
*T*	Absolute temperature [K]
***Subscripts and Superscripts:***	
*f*	Feed
*p*	Permeate
*b*	Bulk
*m*	Membrane
*bf*	Bulk feed
*bp*	Bulk permeate
*mf*	Feed side of membrane
*mp*	Permeate side of membrane
***Greek Letters:***	
*δ*	Membrane thickness [µm]
*ε*	Porosity [%]
*τ*	Tortuosity [dimensionless number]
*λ*	Mean free path [m]
*µ*	Dynamic viscosity [Ns/m^2^]
*V*	Kinematic viscosity [m^2^/s]
*ρ*	Density [kg/m^3^]
